# Development and characterization of a novel biodegradable and antioxidant film based on marine seaweed sulfated polysaccharide

**DOI:** 10.1002/fsn3.3361

**Published:** 2023-04-17

**Authors:** Maedeh Asad Samani, Sedigheh Babaei, Mahmood Naseri, Marjan Majdinasab, Abdorreza Mohammadi Nafchi

**Affiliations:** ^1^ Department of Natural Resources and Environmental Engineering, School of Agriculture Shiraz University Shiraz Iran; ^2^ Department of Food Science and Technology, School of Agriculture Shiraz University Shiraz Iran; ^3^ Food Technology Division, School of Industrial Technology Universiti Sains Malaysia Penang Malaysia; ^4^ Green Biopolymer, Coatings & Packaging Cluster, School of Industrial Technology Universiti Sains Malaysia Penang Malaysia

**Keywords:** agar, chitosan, cross‐linking, fucoidan, *Gracilaria Corticata*, seaweed

## Abstract

This research aims to produce an antioxidant and biodegradable polysaccharide film by using macroalgae agar and sulfated polysaccharide. Agar and sulfated polysaccharide (fucoidan) were extracted from *Gracilaria corticata* and *Sargassum angustifolium* macroalgae. Five treatments were conducted: (A) agar film (1%, W:V), (C) chitosan film (1%, W:V + 1% acetic acid), (AC) agar:chitosan composite (50:50, V:V), (ACF) AC film with fucoidan (0.5%, W:V), and (ACFA) ACF film with citric acid (30% of the dry weight of film) as a cross‐linking agent. Then, 0.75% (V:V) of glycerol was added to all films. The physical, mechanical, antioxidant, color variations, microstructure (SEM), and Fourier transform infrared (FT‐IR) spectroscopy were investigated. Based on the results, modifying the agar film with chitosan improved the mechanical strength, humidity, and solubility in the AC composite film (*p* < .05). Further, adding sulfated polysaccharide and citric acid cross‐linking agent to the agar–chitosan composite led to a significant decrease in solubility, humidity, and permeability to water vapor in ACFA films (*p* < .05), indicating strong cross‐linking and reduction in film pores based on the SEM pictures and FTIR results. However, the physical and mechanical properties of the agar‐based film obtained from Gracilaria algae can be improved by adding chitosan and citric acid cross‐linking agent, and the addition of fucoidan obtained from Sargassum algae has improved its antioxidant properties. This biodegradable film can be a good candidate for preserving perishable products.

## Graphical abstract

In this research, the feasibility of producing a novel biodegradable film based on marine algae polysaccharide was investigated. The agar was extracted from *Gracilaria corticate* and fucoidan was extracted from *Sargassum angustifolium* macroalgae. In addition, citric acid was used as a cross‐linking agent to improve the physical and mechanical properties of the film.

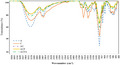

## 1 INTRODUCTION

Today, the approach of using biodegradable food coatings and films is developing because, in addition to maintaining and improving the chemical, organoleptic, and physical properties of food (Bykov et al., 2017; Kocira et al., 2021), the result of biological decomposition of these types of packaging are non‐toxic compounds such as water, carbon dioxide, and mineral compounds (Siracusa et al., 2008).

Edible films and coatings are promising food packaging solution and have gained attention for their ability to break down naturally. They offer advantages such as safety, versatility, and cost‐effectiveness, making them important for sustainable food packaging (Jahdkaran et al., 2021). The use of biodegradable polymers in the production of edible films and coatings is a crucial step toward creating safe and environmentally responsible packaging (Azlim et al., 2022). Edible films and coatings not only act as barrier to preventing the penetration of gases, vapors, and oils but can also carry antimicrobial, antioxidant, and flavoring active agents (Bykov et al., 2017). Biodegradable polymers are divided into three main categories: polysaccharide, protein, and lipid (Zhang et al., 2019). Biodegradable polysaccharide polymers such as cellulose derivatives, chitosan, starch, pectin, carrageenan, and gums (Cazón et al., 2017) have attracted a lot of attention in food preservation due to their low price, abundance in nature, non‐toxicity, and selective permeability to oxygen and carbon dioxide (Mohamed et al., 2020).

Agar is utilized to prepare edible films due to its thermoplastic and biodegradable properties. Agar is considered a type of linear polysaccharide extracted from the cell wall of red marine algae (*Rhodophyceae*), especially *Gelidium* sp. and *Gracilaria* sp. (Zhang & Jiang, 2019). Adding agar to the gelatin film increased the hardness and decreased the permeability of the films to water vapor and solubility, as well as the transmission of ultraviolet rays from the films (Mohajer et al., 2017). However, to improve the mechanical and physical properties of agar, such as fragility and high permeability to water vapor, its combination with other polymers, plasticizers, and nanoparticles can be used (Saurabh et al., 2015; Wang et al., 2018). The previous studies showed that the combination of agar with chitosan improves its mechanical properties due to the hydrogen bond interactions between the amine group of chitosan and the hydroxyl group of agar (Sinaga et al., 2020).

Chitosan is a natural linear polysaccharide that is obtained from chitin in the exoskeleton of crustaceans and the cell wall of fungi during the deacetylation process (Oladzadabbasabadi et al., 2022). Due to the presence of positive functional groups in chitosan, this macromolecule has special characteristics, which can be mentioned as its biodegradability, non‐toxicity, antibacterial, and antifungal activities (Benhabiles et al., 2012). Coating food with chitosan films delayed enzymatic browning, reduced water loss, increased natural flavor, and improved the textural quality of food and color stabilization (Duran & Kahve, 2016). Applying chitosan film in the packaging of rainbow trout burgers (Valizadeh et al., 2019) and chicken fillets (Petrou et al., 2012) increased the shelf life. In addition, adding chitosan with different concentrations to agar films improved the mechanical, antimicrobial, and UV protection properties of agar films (Cao et al., 2018).

Antioxidant compounds can be used in the structure of agar‐based films to prevent food spoilage resulting from its weak antioxidant activity (Wijesekara et al., 2011). Based on the studies, different algae species exhibit natural antioxidant properties due to the presence of fucoidan, carrageenan, ulvan, fucoxanthin, astaxanthin, and various phenolic compounds such as gallic acid, protocatechuic acid, vanillic acid, and quercetin (Ebrahimi et al., 2021; López et al., 2011). Moreover, the cell wall of some brown macroalgae demonstrated higher antioxidant potential compared to red and green macroalgae (Vardizadeh et al., 2021).

Sulfated polysaccharides are useful macromolecules with antioxidant, antiviral, and antibacterial properties in the cell wall of macroalgae (Wang et al., 2010). Although it does not have the ability to form a film by itself, adding it to other films can be beneficial. Which can be beneficial in combination with other films. Fucoidan polysaccharide is regarded as a non‐toxic and biocompatible compound introduced by the Food and Drug Administration (FDA) as a safe food ingredient (Citkowska et al., 2019). The edible film based on chitosan with alginate and fucoidan from seaweed displayed high antioxidant properties (Gomaa et al., 2018). Adding fucoidan extracted from brown macroalgae to alginate/chitosan films increased the thickness and antioxidant properties and decreased the solubility of the film (Gomaa et al., 2018). Further, adding fucoidan to fish gelatin films raised the thickness, tensile strength, elasticity, and elongation at the break of the films (Govindaswamy et al., 2018). Thus, adding fucoidan to agar/chitosan film appears to improve some physical and mechanical properties of the film in addition to its antioxidant properties.

One polymer modification technique is creating cross‐links within or between molecules. Cross‐linking agents cause transverse connections of polymer chains by covalent or non‐covalent bonds and create three‐dimensional (3D) networks, which improve mechanical properties, resistance to water vapor, and reduce film solubility and swelling (Azeredo & Waldron, 2016). Polymers with a high degree of cross‐linking exhibit a harder structure, resulting in trapping more active agents, such as antioxidants, and controlling their release (Khalid et al., 2009). Citric acid is considered a non‐toxic and inexpensive carboxylic acid, widely utilized as an ionic and covalent cross‐linking agent in the food and pharmaceutical industries to make edible polysaccharide films and is considered safe for food use due to its biodegradability (Khouri, 2019). Applying citric acid cross‐linking agents increased antimicrobial properties and resistance to water penetration in starch/chitosan films (Wu et al., 2019). In addition, adding citric acid to the starch film caused a significant decrease in the permeability of the film to water vapor, which is probably due to the creation of a tortuous path by the cross‐linking agent for the transfer of water molecules through the matrix (Ghanbarzadeh et al., 2011).

Therefore, in this study, while investigating the feasibility of producing a biodegradable film from algae agar *Gracilaria corticate* and chitosan, the effect of fucoidan extracted from *Sargassum angustifolium* macroalgae on the antioxidant efficiency of the film was investigated. In addition, citric acid was used as a cross‐linking agent to improve the physical and mechanical properties of the film.

## 2 MATERIALS AND METHODS

### 2.1 Materials

Acetone, 2,2‐diphenyl‐1‐picrylhydrazyl (DPPH), CaCl_2_, ethanol (96%), citric acid, acetic acid, chitosan (MW 450 kDa, 75–85 deacetylated), glycerol, magnesium nitrate, etc were used. All chemicals, reagents, and standards with premium quality were obtained from Sigma–Aldrich, Zakaria Tajhiz Parseh, and Merck.

### 2.2 Preparation of macroalgae

*Gracilaria corticata* and *Sargassum angustifolium* (from the coast of the Persian Gulf) were obtained from the Fars Science and Technology Park, Abdf company‐algae bank, Shiraz, Iran. Macroalgae were first cleaned and then washed several times using fresh water. They were dried in an oven at 38°C for 24–48 h and then powdered and stored at −20°C (Ebrahimi et al., 2021).

### 2.3 Extracting agar from Gracilaria algae

In order to extract agar, 500 mL of distilled water and acetic acid 0.5% were added to 50 g of algae *G. corticata* to reach pH 4.6. The algae were washed with water after stirring for 1 hour until the pH was completely neutral. Then, 1000 mL of distilled water was added, and the mixture was autoclaved at 120°C for 1 hour. In the next step, algae were filtered utilizing a cleaning cloth. The achieved liquid was converted into a gel after cooling at room temperature. In the next procedure, the gels were cut and frozen at −20°C for 24 h. The agar pieces were washed with distilled water after defrosting. Finally, the mixture was dried and powdered in an oven at 60°C and stored in a glass container for later use (Vuai & Mpatani, 2019).

### 2.4 Extraction of SPs from Sargassum

Thirty grams of dry powder of Sargassum was mixed with ethanol 80% in a ratio of 1:20 (w: v) and shacked at ambient temperature overnight. Then, the supernatant was separated using a Buchner funnel. The alga precipitate was bleached with acetone, mixed with distilled water (1:20; w:v), and incubated at 65°C for 3–4 h. After centrifugation (Centrifuge, K241R, Benchtop Centrifuges) in 5000 *g* for 20 min, the supernatant was concentrated in a rotary evaporator (Fara Azma, Iran) at 65°C. The supernatant was mixed with CaCl_2_ (1%, w:w) (to remove the alginate) and kept in the refrigerator overnight. After centrifugation (5000 *g* for 10 min at ambient temperature), cold ethanol 96% (1:3, v:v) was added to the supernatant and kept under refrigerated (4°C) overnight. The SPs were centrifuged and washed with ethanol 96% and acetone and then dried (Bahramzadeh et al., 2019; Vardizadeh et al., 2021).

### 2.5 Preparing agar film and chitosan

To conduct the study, five treatments, including agar film (A), chitosan (C), agar/chitosan (AC) composite, composite with fucoidan (ACF), and composite/fucoidan with the citric acid cross‐linking agent (ACFA) were examined.

In order to prepare agar film (A), 1 g of agar was dissolved in 100 mL of distilled water at a temperature of 90°C on a heteromagnet to produce a homogeneous solution of agar. Then, 1 g of chitosan (C) was dissolved in 100 mL of distilled water and 1% acetic acid at a temperature of 60°C on a heteromagnet to generate a homogeneous solution of chitosan. In the next step, 0.75% (volume/volume) of glycerol was added to agar and chitosan solution separately as a softener, and 15 mL of the solutions was added on a flat surface to plates with a diameter of 10 cm. Finally, agar and chitosan films were dried at 40°C in an incubator for 24 h (Cao et al., 2018; Sinaga et al., 2020).

### 2.6 Preparing agar/chitosan composite film with fucoidan and citric acid

To prepare agar/chitosan composite film (AC), 1% chitosan solution with a ratio of 50:50 was added to 1% agar solution drop by drop. Then, 0.75% (volume/volume) of glycerol was added to the mixture as mentioned earlier and placed on a stirrer at 60°C with 600 rpm for 60 min. In the next step, 15 mL of the composite solution was poured into 10 cm plates and dried in an incubator at 40°C for 24 h. In the next procedure, ACF treatment was prepared by adding 0.5% fucoidan (W:V) to the agar/chitosan composite solution and dried according to the aforementioned steps (Govindaswamy et al., 2018; Yuan et al., 2021). In order to prepare ACFA film, 30% of dry weight (agar and chitosan) citric acid was added to the agar/chitosan/fucoidan solution. Finally, the mixture was homogenized on a stirrer for 30 min and dried in the pellet in the incubator based on the above‐mentioned steps (Uranga et al., 2020; Wu et al., 2019).

### 2.7 Scanning electron microscopy (SEM)

The microstructure of films was evaluated using a scanning electron microscope (TESCAN vega3, Czech Republic) at an accelerating voltage of 20 kV. The microstructure photos of the surface and the cross‐section of the films were taken. The films were mounted on the sample holder with aluminum tape and then sputtered with gold (Desk Sputter Coater, DSR1, Nanostructural Coating Co.).

### 2.8 Fourier transform infrared (FT‐IR) spectroscopy

The interaction of functional groups in different films was evaluated by TENSOR II FT‐IR Spectrometer (Bruker). All infrared spectra were recorded at room temperature (25 ± 1°C) in absorbance from 4000 to 400 cm^−1^ wavenumber.

### 2.9 Mechanical properties of films

The tensile strength (TS, MPa) and elongation at break (EB, %) of each film were determined according to the ASTM standard method D882‐97 (ASTM, 2002) using a testing machine (TA‐XT2, Stable Micro System Ltd.), as described by Mousavi et al. (2022) in details. The film was cut into rectangles strips (10 × 60 mm) and kept in a desiccant containing magnesium nitrate at 51 ± 2% relative moisture and 25 °C for 24 h before the test. The speed of the mechanical crosshead was set at 50 mm/min, while the initial grip separation was set at 40 mm. Three measurements were carried out for each treatment.

### 2.10 Physical properties of films

#### 2.10.1 Film thickness

Physical tests include film thickness, film moisture, water solubility, and water vapor permeability. This was done according to the following methods: The film thickness was determined using a manual digital micrometer (Mitutovo No. 293‐766) with a precision of 0.001 mm. Reported values were average of at least five random locations for each film (Ballesteros et al., 2018).

#### 2.10.2 Film moisture

A certain amount of samples was weighed and then dried for 24 h at 105°C in an oven (T23A, Iran Azuma) until a constant weight was reached. Three replications of each treatment were calculated as below:

$$\text{Moisture content}\%=\left(\text{Initial weight}-\text{Final weight}/\text{Initial weight}\right)\times 100$$

#### 2.10.3 Water solubility

The film water solubility was determined according to the method described by Ballesteros et al. (2018). The oven‐dried film pieces (2 × 2 cm^2^) were immersed in 50 mL of distilled water for 24 h at 25°C in a shaker incubator, and the remnant of the film was finally dried at 105°C. The solubility percentage was obtained from the following formula:

$$\text{Solubility}\%=\left(\text{Initial weight}-\text{Final weight}/\text{Initial weight}\right)\times 100$$

#### 2.10.4 Water vapor permeability (WVP)

The WVP of films was determined according to the standard procedure ASTM E96 (2000) described by Valizadeh et al. (2019) and Mousavi et al. (2021). The circular test cups (3 × 3 × 4 cm) containing 4 g calcium chloride (CaCl_2_) desiccant (0% RH, assay cup) and a control cup (empty of CaCl_2_) were sealed by the prepared films. The cups were placed in a supersaturated NaCl desiccator (RH 75%). The difference in RH corresponds to a driving force of 1753.55 Pa, expressed as water vapor partial pressure. Changes in the weight of the cups (with hourly intervals) were measured and plotted as a function of time. The weight gain versus time plot slope was divided by the exposed film area to obtain the water vapor transmission rate (WVTR). This was multiplied by the thickness of the film and divided by the pressure difference between the inner and outer surfaces to obtain the WVP. All tests were made in triplicate.

$$\mathrm{WVP}=\text{WVTR}\times \text{thickness}\;\left(\mathrm{mm}\right)/A\left(P1-P2\right)$$

WVTR = line slope, P1 = Outside pressure of the cup, P2 = Vapor pressure inside the cup and film, and *A* = surface of cups (mm^2^).

### 2.11 Color measurement

Color properties of films were evaluated in a smart colorimeter (MAT, 2000 Series. IDME Co., Ltd.) based on CIELab scales, in which L* denotes lightness, a* was the representative of redness or greenness, and b* indicated yellowness or blueness. Total color change (Δ*E*) and whiteness index (WI) were calculated using the following equations (Ghafoori Ahangar et al., 2018; Li, et al., 2021):

$$100-\sqrt{{\left(100-L^{*}\right)}^{2}+a^{*2}+b^{*2}}=\mathrm{WI}$$

$$100-\sqrt{{\left(100-L^{*}\right)}^{2}+a^{*2}+b^{*2}}=\mathrm{WI}$$

### 2.12 DPPH free radical scavenging activity

In order to determine the antioxidant potential of composite films, the DPPH free radical scavenging activities were measured according to Wang et al.'s (2009) method. First, 25 mg of different films were soaked in 5 mL of distilled water. Then, 0.5 mL of methanolic solution of DPPH was added to the 1.5 mL of film extract solution. The resulting solution was shaken and kept under darkness at 25°C for 30 min. Finally, the absorbance was read at 517 nm wavelength using a spectrophotometer (T7, PG Instruments Limited). All parameters were tested in triplicates, and the inhibitory percentage was obtained according to the following equation:

$$\text{Inhibitory}\;\left(\%\right)=\frac{\text{Absorbance of control}-\text{Absorbance of sample}}{\text{Absorbance of control}}\times 100$$

### 2.13 Statistical analysis

Data analysis of different films was carried out using one‐way ANOVA with Duncan's multiple‐range test at a 0.95 level of probability after investigating the normal distribution (Kolmogorov–Smirnov test) and homogeneity of variance using IBM SPSS Statistics version 21 software. All parameters were tested in triplicate.

## 3 RESULTS AND DISCUSSION

### 3.1 Scanning electron microscopy

**FIGURE 1:**
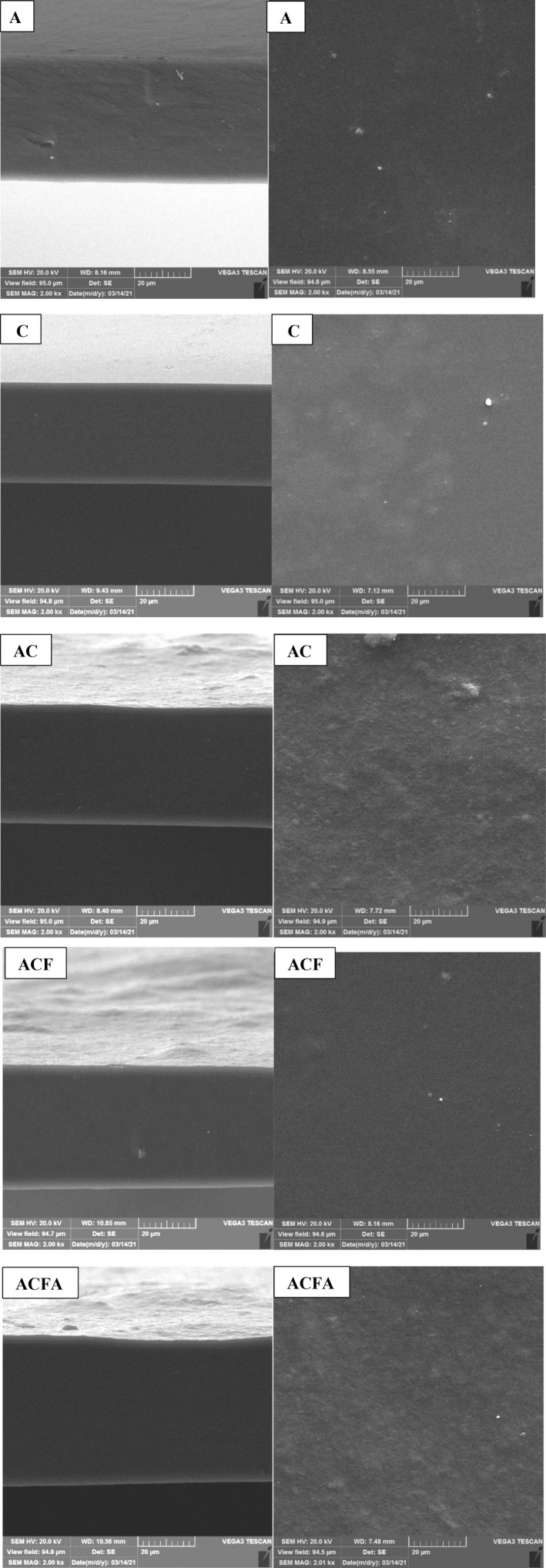
The cross‐sectional (left micrographs) and the surface (right micrographs) SEM micrographs of A (Agar from Gracilaria), C (Chitosan), AC (Agar–Chitosan (Composite)), ACF (Composite + Fucoidan from Sargassum), and ACFA (Composite + Fucoidan + Citric acid as a cross‐linking agent).

Investigating the morphological characteristics of the prepared films is considered an appropriate method to check the homogeneity and uniformity of the prepared composites. Reviewing the microscopic images prepared from the surface of the AC composite displayed a relatively uneven surface compared to the films of agar (A) and chitosan (C) alone (Figure 1), while the cross‐section in all of the films demonstrates a homogeneous and coherent structure. As shown, adding fucoidan to the agar–chitosan composite increases the homogeneity, modifies the film structure, and creates a homogeneous and regular surface and cross‐sectional images of the ACF film. The presence of hydroxyl functional groups in the structure of fucoidan and the compatibility of such compounds with those of polysaccharide films can lead to more connections in the film structure and the formation of homogeneous films. Similar results were observed regarding films prepared from starch and agar (Jumaidin et al., 2016). The surface structure of the film became similar to that of the AC composite by adding citric acid in ACFA films, while the cross‐sectional image of the film was still coherent and homogeneous. Similar results were reported regarding the cross‐sectional image of potato starch/chitosan films with the citric acid cross‐linking agent (Wu et al., 2019). The results of FTIR analysis can prove an increase in hydrogen bonding between film compounds.

### 3.2 FTIR spectroscopy

**FIGURE 2:**
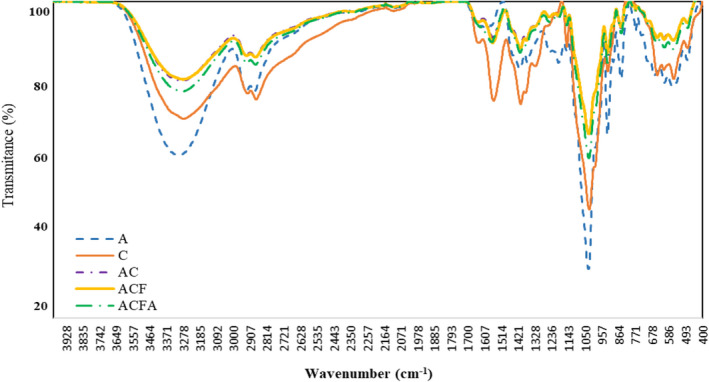
FTIR spectrum of A (Agar from Gracilaria), C (Chitosan), AC (Agar–Chitosan (Composite)), ACF (Composite + Fucoidan from Sargassum), and ACFA (Composite + Fucoidan + Citric acid as a cross‐linking agent).

As illustrated in Figure 2, the peaks observed at the wavelength of 1029 cm^−1^, 1000–1100 cm^−1^, and 1400–1500 cm^−1^ are related to the stretching vibrations of C–O and C–O–C, as well as C–O and C–N functional groups, and the first and second type amino stretching vibrations. The presence of a peak in the range 2800–3000 cm^−1^ can be attributed to the presence of C‐H functional group. In addition, the peak observed at the range 3200–3600 cm^−1^ is related to hydroxyl (–OH) and amine (–NH2) functional groups (Chen et al., 2016). The peaks observed at 1319 and 1405 are related to the pyranose rings in the chitosan structure (Elhefian, 2011). According to Sinaga et al. (2020), the prepared agar–chitosan film also showed similar functional groups in FTIR analysis. Some peaks observed in the regions of 1160–1260 and 845 cm^−1^ can be attributed to the existence of S=O and C‐O‐S groups, respectively (Barbosa et al., 2019). In addition, Vardizadeh et al. (2021) attributed the bands in the range 668–1418 nm to the existence of S=O=S, C=O, and O=S=O groups in sulfated polysaccharides of algae. Adding fucoidan to agar/chitosan films due to an increase in hydroxyl functional groups and the rise in the number of hydrogen bonds may lead to the homogeneity of the ACF film in the scanning electron microscopy (SEM) microscopic images. Adding citric acid as a cross‐linking agent leads to a slight increase in the intensity of the peaks related to hydroxyl (–OH) and amine (–NH2) functional groups in ACFA compared to ACF and AC films, indicating the role of such functional groups in creating crosslinks.

### 3.3 Mechanical properties

#### 3.3.1 Tensile strength or mechanical tension

**FIGURE 3:**
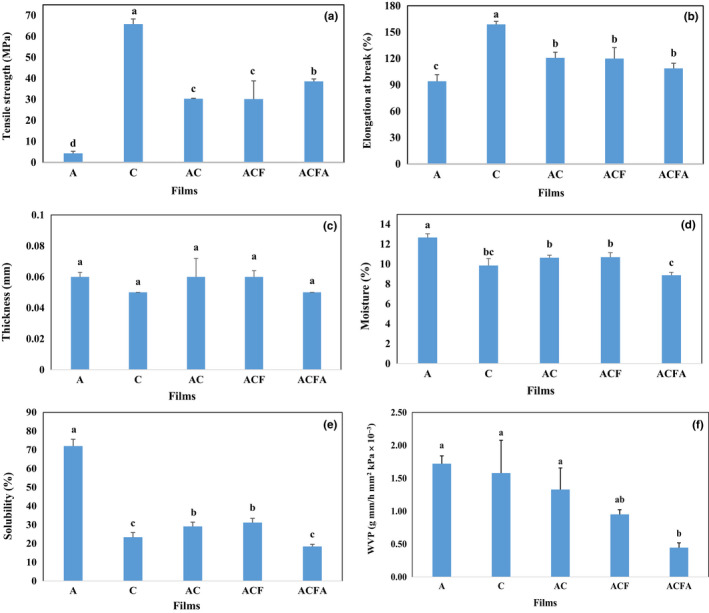
Physical and mechanical characteristics of agar–chitosan composite films incorporated with various additives. A (Agar from Gracilaria), C (Chitosan), AC (Agar–Chitosan (Composite)), ACF (Composite + Fucoidan from Sargassum), and ACFA (Composite + Fucoidan + Citric acid as a cross‐linking agent). Small letters represent a significant difference between different treatments (*p* < .05).

Tensile strength is among the significant parameters studied during preparing the films, indicating their resistance against tearing when exposed to external forces. Figure 3a demonstrates the results related to investigating the tensile strength in the prepared films. The lowest and highest amount of tensile strength belongs to agar and chitosan films (65.8 MPa), respectively (*p* < .05). Chitosan is an alkaline polymer insoluble in pure water and organic solvents, dissolving in acidic organic solutions with a pH of less than 6. Different kinds of organic and inorganic acids, such as acetic, lactic, citric, and hydrochloric acids, can dissolve chitosan (Melro et al., 2021) and affect the mechanical properties of chitosan films (Nadarajah, 2005). Based on the studies, adding chitosan to agar film improves its mechanical properties by creating hydrogen bonds between agar and chitosan (Sinaga et al., 2020), resulting in modifying AC composite and increasing its tensile strength compared to agar film. Modifying ACF composite with citric acid increased the tensile strength of ACFA films. Gan et al. (2018) reported that adding citric acid to the cellulose polymer composite and nanocrystalline chitosan increased the stability of the existing network between the cross‐linking agent and the film matrix (Gan et al., 2018). In addition, Melro et al. (2021) indicated that the films containing citric and lactic acids displayed the highest and lowest tensile strength, respectively, in the use of different organic acids as cross‐linking agents in films. Generally, applying a cross‐linking agent improves mechanical properties such as the degree of swelling and tensile strength in addition to creating new chemical bonds and increasing internal connections between polymer chains. The resistance to deformation and disintegration of the film increases in chitosan films together with the citric acid cross‐linking agent (Pavoni et al., 2021).

#### 3.3.2 Elongation percentage at the breaking point

As Figure 3b displays, agar and chitosan films exhibit the lowest and highest elongation at the breaking point, which is almost in line with the tensile strength results. As observed, adding chitosan to the agar film improves the AC film compared to the first one, indicating a similar result in the tensile strength (Figure 3a). Sinaga et al. (2020) argued that the lowest elongation percentage is related to the agar film, and adding glycerol increases such a parameter from 0.2% to 9.8%. Based on the results, adding chitosan to agar/glycerol film increased the elongation percentage to about 19% (Sinaga et al., 2020). Moreover, the mechanical properties of chitosan films in combination with some compounds such as polyethylene oxide (PEO) exhibit a slight decrease. It was reported in the connection between chitosan and PEO, resulting in decreasing the tensile strength and elongation percentage in the chitosan/polyethylene oxide composite film (Zivanovic et al., 2007). Further, the combined chitosan/polyvinyl alcohol film displayed intermediate mechanical properties compared to the films mentioned earlier alone (Costa‐Júnior et al., 2009). Adding fucoidan and citric acid did not affect the percentage elongation in ACF and ACFA films significantly. The cross‐linking agent reduces the mobility of molecules and polymer chains by creating crosslinks and decreases the elongation percentage, resistance to water vapor, solubility, and swelling in films (Azeredo & Waldron, 2016; Liang et al., 2019; Mrsny, 1992). However, Liang et al. (2019) claimed that different cross‐linking agents demonstrate diverse elongation in the prepared films.

### 3.4 Physical properties of the film

#### 3.4.1 Thickness

As shown in Figure 3c, no significant difference is observed in the thickness of different films (*p* < .05). Elhefian (2011) asserted that an increase in the amount of agar in the preparation of agar/chitosan films did not affect the thickness of the films significantly. Similar results were reported regarding the thickness of the films prepared by combining gelatin and starch (Ma et al., 2021), those made of soy protein, starch, and maltodextrin (Galus et al., 2012), and kappa‐carrageenan films with a savory essence (Shojaee‐Aliabadi et al., 2013), indicating appropriate compatibility between film components. The studies as mentioned earlier indicated that the difference in the thickness of the films depends on the preparation method and the amount of additive. Utilizing the cross‐linking agent leads to a slight decrease in the thickness of the ACFA film, probably due to the creation of strong intermolecular interactions between chitosan and agar. The cross‐linking agent can reduce the film thickness by decreasing the viscosity in the initial film solution (Li, 2008).

#### 3.4.2 Humidity

Figure 3d illustrates the results related to the moisture content in different films. As observed, agar and ACFA films exhibit the highest (12.7 ± 0.4%) and lowest (8.9 ± 0.3%) moisture levels (*p* > .05), respectively, while no significant difference is observed between the moisture content in other films (*p* < .05). Combining chitosan with agar film adjusts the moisture content in AC composite. Such difference in the amount of water absorption in agar and chitosan can be related to the presence of more hydroxyl groups in the agar structure (Elhefian, 2011). Adding fucoidan to AC composite did not affect moisture content significantly (*p* < .05) while adding citric acid as a cross‐linking agent reduced the moisture content in ACFA film by about 17% compared to ACF film. Creating hydrogen bonds between hydroxyl and amine groups in the structure of polymers applied in the composite reduced the access of water molecules to such functional groups and decreased film humidity (Alvarado et al., 2015). In addition, assessing the electron microscope results displayed the creation of a highly homogeneous structure in the cross‐section of the film prepared in the presence of the cross‐linking agent, indicating the reduction of the pores in the films and their moisture (Gomes et al., 2019).

#### 3.4.3 Solubility

Water sensitivity is among the most significant factors limiting the use of polysaccharide films in food packaging. Generally, films with a high solubility percentage are utilized for preparing food coatings and linings, while those with a low solubility percentage are applied for food packaging (Gomaa et al., 2018). As demonstrated in 3e, the solubility check results are consistent with the moisture content in the films. Agar film displays high solubility, while chitosan one shows little solubility in water, similar to previous studies (García et al., 2004). In fact, chitosan is soluble in dilute acidic solution with a pH of less than 6.5 and insoluble in pure water (Jin & Song, 2006). Adding chitosan to the agar film improved and reduced the solubility of AC film by 40% compared to A (*p* > .05), while no significant difference in solubility was reported by adding fucoidan to AC film (*p* < .05). Using cross‐linking agent in the formation of films reduced the solubility of the film from 31.15 (ACF) to 18.41% (ACFA), which may be due to an increase in creating links (*p* > .05; Figure 3e). The controlled solubility of biodegradable films exhibits a large number of advantages in the pharmaceutical, agricultural, and food industries, although the high resistance of plastic films to water is regarded as critical in different industries. For example, microporous chitosan/polyethylene oxide membranes can control the drug release rate by gradually dissolving polyethylene oxide in water and changing the membrane size (Jin & Song, 2006).

#### 3.4.4 Water vapor permeability

Water vapor permeability (WVP) is regarded as a measure that demonstrates the ease of water vapor penetration and the ability of film to control the transfer of water vapor between the food system and the environment (Li, 2008). Figure 3f shows the results related to the permeability of the prepared films. As observed, agar and chitosan films and their composite display high permeability against water vapor. Generally, chitosan films create a relatively weak barrier against moisture, resulting in limiting their use in food packaging (Aydinli & Tutas, 2000). The results indicated that adding citric acid led to a four‐fold decrease in the permeability of ACFA composite (0.45 ± 0.07 g mm/h mm^2^ kpa × 10^−6^) (*p* > .05). Such reduction in permeability by adding a cross‐linking agent resulted from increasing the interactions between chitosan and agar polymers and creating a barrier against water vapor. Based on the results, the change in the intensity of the peaks related to the functional groups due to the addition of fucoidan and the cross‐linking agent can be attributed to the creation of hydrogen bonds and likely prevents the permeability of water vapor in the films. Davoodi et al. (2021) declared that films prepared to utilize sulfated polysaccharides extracted from marine green algae *ulva intestinalis* show high permeability to water. However, adding sulfated polysaccharide from Sargassum to the ACF film decreased the amount of WVP. Factors such as adding softening materials, film manufacturing methods, and experimental conditions such as relative humidity, temperature, and film thickness affect the permeability of the film (Li, 2008). However, reports indicated that some compounds, such as plant polyphenols in tea extract, can increase moisture resistance by creating hydrogen and covalent bonds with chitosan (Kumar et al., 2020). Moreover, the interaction between negatively charged agar and positively charged chitosan due to low pH (employing acetic acid for the dissolution of chitosan and citric acid as cross‐linking agents) can affect the film integrity, resulting in decreased WVP.

### 3.5 Color analysis

The transparency of edible films plays a critical role in their acceptability. As Table 1 indicates, film color changes (Δ*E*) are evaluated by applying *L* (clarity), *a* (red–green), and *b* (blue–yellow) parameters. As observed, adding fucoidan to the combined film of ACF and ACFA changes ΔE and whiteness indices (WI) significantly (*p* > .05), while adding citric acid to the film does affect the color of the samples significantly. In addition, no significant difference was observed between A and AC and C films. Adding turmeric extract to chitosan films decreased the WI and changed the color to yellow. Furthermore, a significant difference was reported in the color change index in films containing fennel and cinnamon extracts (Liu et al., 2021). In another study, adding purple tomato anthocyanin as an antioxidant to chitosan film led to significant changes in the color change index (Li et al., 2021). Ojagh et al. (2010) and Valizadeh et al. (2019) had shown that the addition of cinnamon essential oil and oleic acid was the main reason for color change in the chitosan‐based films. However, the results of this study and mentioned studies showed that the color and transparency of edible films and coatings are greatly influenced by the compounds that are used in them, and it is not a reason for reducing or increasing their efficiency. The transparent films should be used in some food products, and the actual color of the product cannot be observed due to the turbidity of the prepared film or coating. However, opaque films can be used in dairy products, pharmaceuticals, or light‐sensitive products (Bharti et al., 2021).

**TABLE 1:** Color change (Δ*E*), L* index, a* index, b* index, and yellowness index (WI) of agar–chitosan composite films incorporated with various additives.

Treatments	L*	a*	b*	Δ*E*	WI
A	62.42 ± 2.05^ab^	−6.41 ± 0.18^ab^	7.19 ± 0.47^c^	Control	61.21 ± 1.18^ab^
C	65.49 ± 2.65^a^	−5.45 ± 0.14^a^	3.22 ± 0.37^d^	5.48 ± 1.03^c^	64.92 ± 1.52^a^
AC	62.87 ± 4.02^ab^	−8.45 ± 0.45^c^	15.39 ± 0.43^b^	9.38 ± 0.23^b^	58.91 ± 2.22^b^
ACF	58.04 ± 2.29^ab^	−7.67 ± 0.62^bc^	22.15 ± 1.45^a^	15.86 ± 0.49^a^	51.87 ± 0.7^c^
ACFA	56.27 ± 2.72^b^	−7.6 ± 0.64^bc^	22.09 ± 0.64^a^	16.41 ± 0.27^a^	50.38 ± 1.67^c^

*Note*: A (Agar from Gracilaria), C (Chitosan), AC (Agar–Chitosan (Composite)), ACF (Composite + Fucoidan from Sargassum), and ACFA (Composite + Fucoidan + Citric acid as a cross‐linking agent). Small letters represent a significant difference between different treatments in each column (*p* < .05).

### 3.6 Antioxidant properties

**FIGURE 4:**
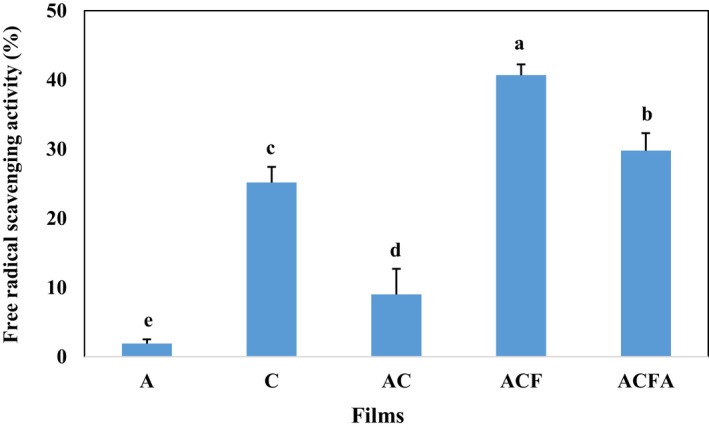
Radical scavenging activity of agar–chitosan composite films incorporated with various additives. A (Agar from Gracilaria), C (Chitosan), AC (Agar–Chitosan (Composite)), ACF (Composite + Fucoidan from Sargassum), and ACFA (Composite + Fucoidan + Citric acid as a cross‐linking agent). Small letters represent a significant difference between different treatments (*p* < .05).

Figure 4 illustrates the results related to the antioxidant activity of the prepared films. As observed, agar shows the lowest, and chitosan displays a higher antioxidant activity, moderated in AC film (*p* > .05). Chitosan extracted from shrimp can inhibit DPPH free radicals, deter hydrogen peroxide radicals, and reduce trivalent iron cation (Tanha et al., 2017). The results indicated that chitosan oligomers inhibit intracellular free radicals, myeloperoxidase enzyme in myeloid cells, oxidized proteins, and DNA in macrophage cells (Ngo & Kim, 2014). In addition, chitosan films can prolong the shelf life and increase the nutritional value of food combined with other substances such as flavors, colors, antioxidants, and antimicrobial agents such as essential oils (Li, 2008). Based on the results, adding a cross‐linking agent to the film leads to a slight decrease in the antioxidant property of ACFA film compared to ACF one. Similar results showed adding glutaraldehyde as a cross‐linking agent in gelatin–chitosan composite film decreased the antibacterial effect of ε‐poly‐L‐lysine (Mousavi et al., 2021). Actually, the dense and compact microstructure created by cross‐linking agents limited the antioxidant or antibacterial compounds release from the film, and consequently decreased their performance. The highest level of antioxidant property was reported in the film enriched with fucoidan (ACF) (*p* > .05). Some studies indicated that sulfated polysaccharides and extracts from Sargassum algae exhibit significant antioxidant properties due to their sulfated functional groups and high phenol (Ebrahimi et al., 2021; Mousavipour et al., 2021; Vardizadeh et al., 2021). The effectiveness of antioxidant activity in films combined with plant flavonoids has been reported frequently, among which applying grape seed, pomegranate peel, apple peel, and thyme extracts is considered the most significant one (Kumar et al., 2020).

## 4 CONCLUSION

Based on the results, modifying the agar film obtained from Gracilaria algae with chitosan improves the mechanical strength, humidity, and solubility in the AC composite film. Further, adding sulfated polysaccharide extracted from Sargassum algae and citric acid cross‐linking agent to the agar–chitosan composite leads to a significant decrease in solubility, humidity, and permeability to water vapor in ACFA films, indicating strong cross‐linking and reduction in film pores. Finally, adding sulfated polysaccharide increases the antioxidant activity in the films, indicating that such biodegradable film can be proposed as an appropriate candidate for use in preserving food and marine products.

## AUTHOR CONTRIBUTIONS

**Maedeh Asad Samani:** Investigation (equal); methodology (equal); validation (equal); writing – original draft (equal). **Sedigheh Babaei:** Project administration (equal); software (equal); supervision (equal); validation (equal); writing – original draft (equal); writing – review and editing (equal). **Mahmood Naseri:** Conceptualization (equal); data curation (equal); methodology (equal); resources (equal); software (equal). **Marjan Majdinasab:** Formal analysis (equal); methodology (equal); resources (equal); visualization (equal). **Abdorreza Mohammadi Nafchi:** Conceptualization (equal); formal analysis (equal); validation (equal); writing – review and editing (equal).

## CONFLICT OF INTEREST STATEMENT

The authors declare that they have no known competing financial interests or personal relationships that could have appeared to influence the work reported in this paper.

## Data Availability

The data that support the findings of this study are available from the corresponding author upon reasonable request.
